# Comparison of Semi-Automated and Manual Measurements of Carotid Intima-Media Thickening

**DOI:** 10.1155/2014/531389

**Published:** 2014-01-21

**Authors:** Oscar Mac Ananey, Greg Mellotte, Vincent Maher

**Affiliations:** Department of Cardiology, Tallaght Hospital, Dublin 24, Ireland

## Abstract

Carotid intima-media thickening (CIMT) is a marker of both arteriosclerotic and atherosclerotic risks. Technological advances have semiautomated CIMT image acquisition and quantification. Studies comparing manual and automated methods have yielded conflicting results possibly due to plaque inclusion in measurements. Low atherosclerotic risk subjects (*n* = 126) were recruited to minimise the effect of focal atherosclerotic lesions on CIMT variability. CIMT was assessed by high-resolution B-mode ultrasound (Philips HDX7E, Phillips, UK) images of the common carotid artery using both manual and semiautomated methods (QLAB, Phillips, UK). Intraclass correlation coefficient (ICC) and the mean differences of paired measurements (Bland-Altman method) were used to compare both methodologies. The ICC of manual (0.547 ± 0.095 mm) and automated (0.524 ± 0.068 mm) methods was *R* = 0.74 and an absolute mean bias ± SD of 0.023 ± 0.052 mm was observed. Interobserver and intraobserver ICC were greater for automated (*R* = 0.94 and 0.99) compared to manual (*R* = 0.72 and 0.88) methods. Although not considered to be clinically significant, manual measurements yielded higher values compared to automated measurements. Automated measurements were more reproducible and showed lower interobserver variation compared to manual measurements. These results offer important considerations for large epidemiological studies.

## 1. Introduction

Vascular risk assessment has become integral to good clinical practice. Conventional risk factors which are derived from a patient's family and smoking history, blood pressure, and measurement of blood glucose and lipid levels have been used successfully to derive a person's future risk of developing atherosclerotic cardiovascular disease [1, 2]. Measurement of carotid intima-media thickness (CIMT), a marker of atherosclerosis risk, can improve individual risk assessment and quantify pathology and/or drug therapy efficacy [3, 4]. Because it is noninvasive, easy to perform, and highly repeatable, a number of epidemiological studies have adopted CIMT as a surrogate marker of cardiovascular risk [5–7]. Technological advances over the past number of years have improved image acquisition and measurement methods [8]. In tandem with these new methodologies, a number of studies have emerged comparing older manual and newer automated/semiautomated methods [8–12]. However, in some of these previous studies the statistical methods may have been unsuitable or the cohort may have been biased. Our aim was to compare manual and semiautomated methods of measuring CIMT in healthy male and female subjects with very low cardiovascular risk. The rationale for the low risk subjects was to minimise the potential influence of plaque on CIMT measurements. In addition, we examined the intraobserver and interobserver variation of each method.

## 2. Material and Methods

One hundred and twenty-six (68 male and 58 female) subjects were recruited from the general population. The study was approved by Trinity College Dublin Ethics Committee. Written informed consent was obtained from all subjects prior to testing protocols. Subjects were included if they were lifelong never-smokers, free from cardiovascular disease, and normotensive (<140/90 mmHg), had normal lipid profile (LDLc < 4.0 mmol/L), normal fasting glucose (fasting glucose < 6.2 mmol/L), and moderate alcohol intake (male < 21 units per week; female < 14 units per week). Subjects were excluded if they were receiving treatment for or had a history of hypertension, hyperlipidaemia, and diabetes or were taking any medications that affected haemodynamic and/or metabolic responses.

Subjects attended the Cardiovascular Research Unit at Tallaght Hospital. Various anthropometrical measurements were recorded, including height (Seca 202, SECA, UK), weight (Avery E101, Avery, UK), and waist/hip circumference (Creative Health Products, USA).

High-resolution B-mode ultrasound images of the right and left common carotid artery were used to measure carotid intima-media thickness. Patients were scanned in the supine position using 7–12 MHz linear array transducer (Philips HDX7E, Phillips, UK).

CIMT was calculated using both manual (Manual) and semiautomated (Automated; QLAB, Phillips, UK) methods. Manual CIMT measurements were recorded from the far wall at 1 cm, 1.5 cm, and 2 cm intervals proximal to the carotid bulb [13]. Automated measurements were also recorded from the far wall, using the same image, from the identical 1 cm section proximal to the carotid bulb. The carotid bulb was defined as the point where the far wall deviated from the parallel plane of the distal CCA. Mean manual and automated CIMT measurements for the right and left CCA were calculated from three consecutive cardiac cycles [14].

Pearson product-moment correlation coefficient and the intraclass correlation coefficient (ICC) were used to examine the relationship between manual and automated methods [15]. The associations of the differences of the mean of the paired measurements (Bland-Altman method) were used to examine absolute differences between the two methods (MedCalc, Belgium).

The technical error of measurement (TEM) and ICC of ten randomly selected subjects were used to identify intraobserver reproducibility and interobserver reliability of the two methods [15, 16].

An unpaired *t*-test was used to compare gender differences (MedCalc, Belgium). Values are reported as mean ± SD unless otherwise stated.

## 3. Results

Subject characteristics and cardiovascular risk factors are outlined in Table 1. There were 65 male and 54 female subjects with a mean age of 40.5 years. No differences in age, diastolic blood pressure, total cholesterol, and LDLc were observed between genders. However, BMI, systolic blood pressure, triglyceride, and glucose were higher and HDLc was lower in males compared to females (*P* < 0.049).

Pearson correlation demonstrated strong association (*r* = 0.80; *P* < 0.0001) between manual (mean ± SD; 0.547 ± 0.095 mm) and automated (mean ± SD; 0.524 ± 0.068 mm) methods; however, the same association was not observed with ICC (*R* = 0.74).

**Figure 1:**
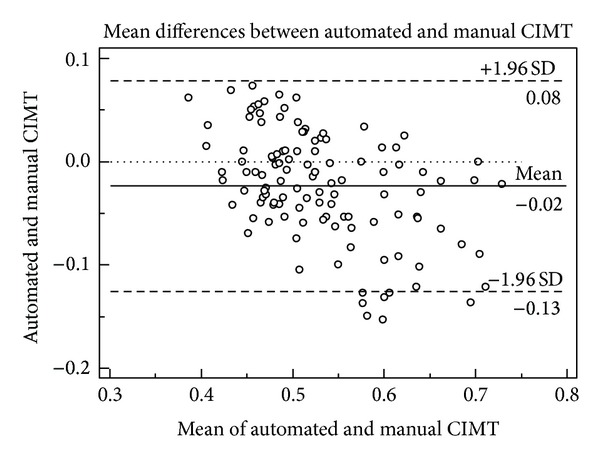
Bland-Altman plot of the absolute mean differences of automated and manual CIMT measurements with a mean bias and SD of −0.023 ± 0.052 mm and limits of agreement of 0.078 to −0.125 mm.

Evaluation of the differences of paired means (Bland-Altman method) identified an absolute mean bias and SD of −0.023 ± 0.052 mm between manual and automated CIMT measurements with limits of agreement of 0.078 to −0.125 mm (Figure 1).

The TEM, quantifying the interobserver reproducibility and intraobserver variability, was lower for automated (3.71% and 1.52%) compared to manual (8.11% and 6.30%) methods. As a consequence, the interobserver and intraobserver ICC was greater for automated (*R* = 0.94 and 0.99) compared to manual (*R* = 0.72 and  0.88) methods.

## 4. Discussion

This study highlights that manual measurements yield higher values compared to automated measurements even in subjects with very low atherosclerotic risk. The mean differences of both methods were not clinically significant and no systematic errors were observed. In the absence of a gold standard measurement such as using a phantom, it is unclear which method best approximates real values.

The results also demonstrate that automated CIMT calculations are more reproducible and show lower interobserver variation compared to manual calculations. These results offer important considerations where patients may be scanned by different technicians and where the accumulation of small variations may impact results, especially in large scale epidemiological studies.

In the present study, Pearson product-moment correlation coefficient demonstrated a strong association between both methods; however, a strong ICC was not observed (*R* < 0.85) [17]. Pearson product-moment correlation coefficient is not considered to be a robust determination of association whereas ICC represents perfect agreement [16]. This was further emphasised by the mean bias of the Bland-Altman plot where manual measurements yielded greater, although not clinically significant, values compared to automated measurements.

Previous studies report no differences between automated versus manual CIMT methodologies whereas other studies report significantly greater values using manual techniques [9, 10, 12].

Seçil et al. [12] reported significantly greater values for manual CIMT calculations compared to automated calculations. The authors reported that manual measurements were significantly higher (1.3–8.7%) compared to automated measurements. In the same study the authors also reported higher interobserver correlation coefficients for automated methods compared to manual methods.

Freire et al. [9] reported no differences between automated and manual CIMT calculations; however automated methods provided lower interobserver and intraobserver variation coefficients. Puchner et al. [10] reported significant correlation (*r* = 0.86; *P* < 0.01) between automated and manual methodologies with no observable systematic bias in the mean differences (mean difference 0.023 ± 0.034 mm). The authors also reported lower interobserver and intraobserver variation coefficients for automated methods (6.6% and 5.6%) compared to manual methods (14.1% and 11.1%). More recently, Yanase et al. [11] reported similar values for manual and automated methods; yet automated calculations had lower standard deviations and variation coefficients indicating better reproducibility. Furthermore, automated methods were better correlated with Framingham and Prospective Cardiovascular Munster study (PROCAM) risk scores.

For the present study, in order to minimise potential measurement inconsistencies caused by abnormal CIMT, focal thickening, or the presence of atheromatous lesions, only subjects with very low cardiovascular risk were recruited. In addition, manual CIMT measurements were averaged from three anatomic sites, over several cardiac cycles from both left and right sides. Despite these precautions, it is possible that outliers may have caused an overestimation of manual CIMT [18]. For automated methods, several hundred measurements are recorded, and so, averaged values would be less susceptible to individual outlier errors [18].

This study does not examine serial changes in CIMT over given time intervals. Such measurements are used in clinical practice as surrogate markers of vascular risk [19]. Larger increments in CIMT are more associated with greater risk of vascular events [20]. However, it is also important to make a clear distinction between changes in CIMT and progression of atheromatous plaque. As atherosclerosis has focal changes more so than uniform changes, variation in CIMT at different segments or changes in maximal CIMT may better represent progression of atherosclerotic disease [18]. Changes in vascular wall properties, characterised by CIMT, represent different disease processes. Standardised definitions of focal plaque structures such as luminal encroachment of 50% or >0.5 mm should be adopted to help differentiate the two distinct diseases processes [18].

Most large scale epidemiological studies have adopted manual methodologies to quantify CIMT with only one study using semiautomated edge detection software [6, 21–23]. Based on the results of our study, it is fair to suggest that future studies, particularly interventional and longitudinal studies, should consider adopting automated CIMT methodologies.

## 5. Conclusion

In conclusion, semiautomated measurements of CIMT yield significantly lower values compared to manual measurements and produce lower intraobserver and interobserver variation. Although the differences between manual and automated methods are small and may not be clinically significant, these observations offer important considerations for large epidemiological or longitudinal studies.

## Figures and Tables

**Table 1:** Subject characteristics and cardiovascular risk factors.

Subject characteristics and cardiovascular risk factors	
(Male *n* = 65; female *n* = 54)	
Age (years)	40.5 ± 10.1
BMI (kg·m^2^)	25.6 ± 4.13
SBP (mmHg)	123 ± 13
DBP (mmHg)	73 ± 8
Total cholesterol (mmol·L^−1^)	4.79 ± 0.76
Triglyceride (mmol·L^−1^)	1.02 ± 0.53
LDLc (mmol·L^−1^)	2.80 ± 0.67
HDLc (mmol·L^−1^)	1.54 ± 0.44
Fasting glucose (mmol·L^−1^)	5.03 ± 0.49

Body mass index (BMI), systolic blood pressure (SBP), diastolic blood pressure (DBP), low-density lipoprotein cholesterol (LDLc), and high-density lipoprotein cholesterol (HDLc). Values are mean ± SD.
